# Author Correction: Aneuploid embryonic stem cells drive teratoma metastasis

**DOI:** 10.1038/s41467-024-53288-0

**Published:** 2024-10-15

**Authors:** Rong Xiao, Deshu Xu, Meili Zhang, Zhanghua Chen, Li Cheng, Songjie Du, Mingfei Lu, Tonghai Zhou, Ruoyan Li, Fan Bai, Yue Huang

**Affiliations:** 1 State Key Laboratory of Common Mechanism Research for Major Diseases, Department of Medical Genetics, Institute of Basic Medical Sciences, Chinese Academy of Medical Sciences, School of Basic Medicine, Peking Union Medical College, Beijing, 100005 China; 2 Biomedical Pioneering Innovation Center (BIOPIC), Beijing Advanced Innovation Center for Genomics (ICG), School of Life Sciences, Peking University, Beijing, 100871 China; 3 Wellcome Sanger Institute, Wellcome Genome Campus, Hinxton, Cambridge CB10 1SA UK; 4 Present Address: Department of Systems Biology, The University of Texas MD Anderson Cancer Center, Houston, TX USA

**Keywords:** Cancer models, Metastasis

Correction to: *Nature Communications* 10.1038/s41467-024-45265-4, published online 5 February 2024

In the original version of this Article there was an error in Figure 6h where the bioluminescent images for Xbp1 in the third and fourth columns were inadvertently duplicated from the Ts11 lung image in Figure 6c and Ts11 lung top-row image Supplementary Fig. 12b, respectively. The Source Data has been updated to include the raw images. This has now been corrected in the PDF and HTML versions of the Article.

## Supplementary information

Revised Source Data

