# Supplementary material for: Aneuploid embryonic stem cells drive teratoma metastasis
Source: Nat Commun. 2024 Feb 5;15:1087. doi: 10.1038/s41467-024-45265-4 (PMC10844504; doi:10.1038/s41467-024-45265-4)
Supplement: Supplementary file 1 — Supplementary Information [file 41467_2024_45265_MOESM1_ESM.pdf]

Supplementary information for

Aneuploid embryonic stem cells drive teratoma metastasis

Authors: Rong Xiao[1†], Deshu Xu[2†], Meili Zhang[1†], Zhanghua Chen[2†], Li Cheng[1], Songjie Du[1], Mingfei Lu[1], Tonghai Zhou[1], Ruoyan Li[3], Fan Bai[2]*, Yue Huang[1]*

*Corresponding author.

Email: fbai@pku.edu.cn (F.B.); huangyue@pumc.edu.cn (Y.H.)

Supplementary information

Supplementary Figures 1-14

Supplementary Tables 1-5

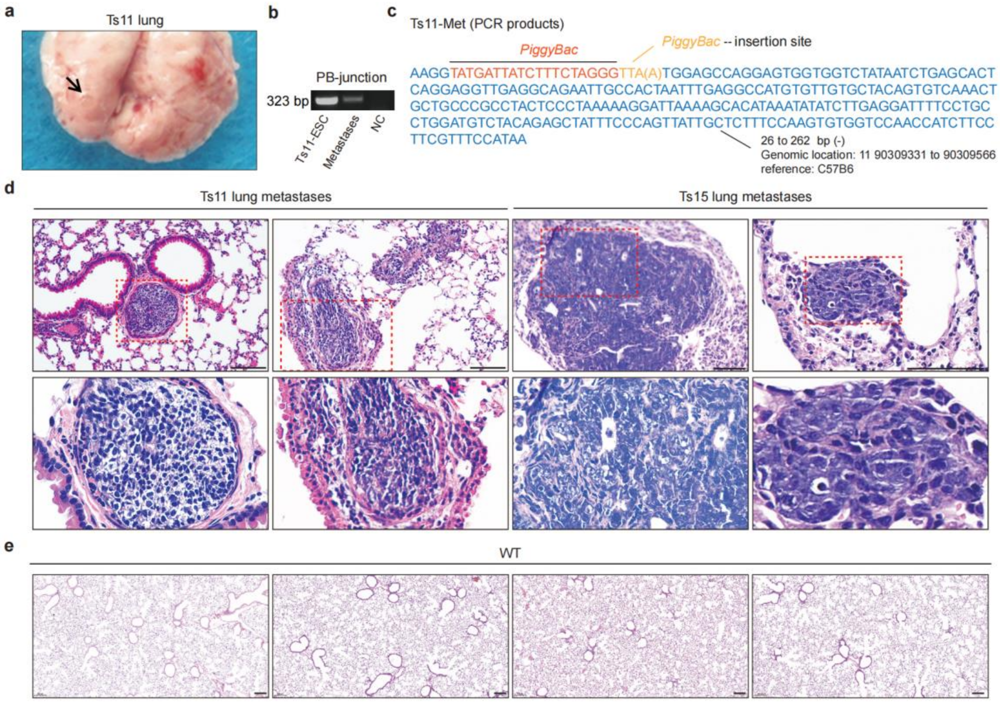

Supplementary Fig. 1 | Metastases were occasionally observed in mice injected with Ts11 or Ts15 ESCs.

a A metastatic nodule observed in the lung of a Ts11 tumor-bearing mouse. The arrow indicates metastasis. **b** Genomic PCR of the unique PB transposon-host junction fragments (PB-junction) confirmed that the lung metastasis originated from the injected Ts11 mouse ESCs. NC, negative control. PB, *piggyBac*. See also Source Data File. **c** NCBI-BLAST results of Ts11-ESCs and Ts11-metastases genomic PCR products of the unique PB transposon-host junction fragments (PB-junction). **d** Histologic architecture of Ts11 and Ts15 lung sections. The lower images are enlarged views of the areas in the red dashed boxes in the upper images. Scale bars, 100 μm. **e** HE staining of lung sections of mice bearing WT teratomas. WT, wild-type.

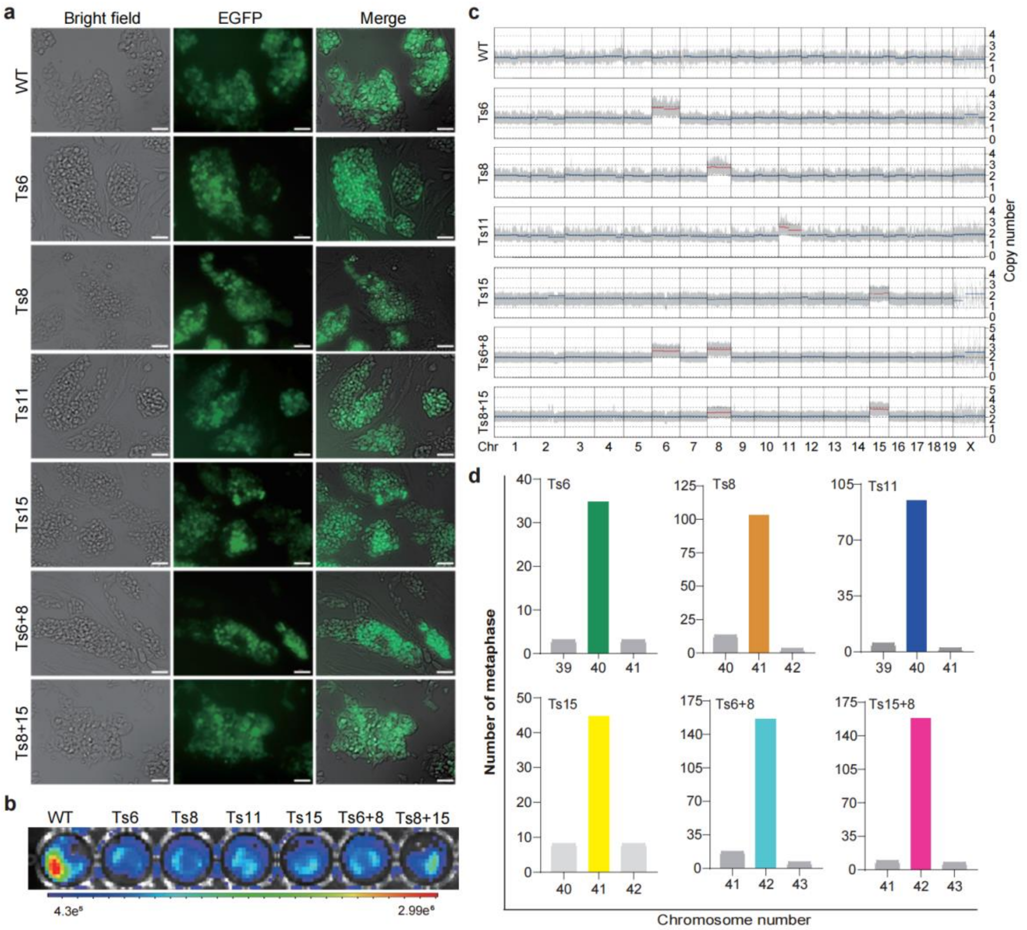

Supplementary Fig. 2 | Establishment of EGFP/luciferase dual-labeled WT and aneuploid mouse ESC lines.

a Bright-field and EGFP images of WT and aneuploid ESCs. Scale bars, 100 µm. **b** Bioluminescent imaging of WT and aneuploid ESCs. The rainbow gradient bar indicates the photon flux. **c** CNV analysis of each aneuploid ESC line. Sequenza was used to analyze WGS data. The gray bars indicate the interquartile ranges. The thick horizontal lines indicate segmented values. Trisomic chromosomes are indicated in red. Chr, chromosome. **d** Distribution of chromosome numbers in each aneuploid ESC line counted by the preparation of chromosome spreads. See also Source Data File. Notably, Ts6 and Ts11 gained an extra copy of chromosome 6 or 11 but lost chromosome Y according to our previous work. Ts, trisomy. See also Source Data File.

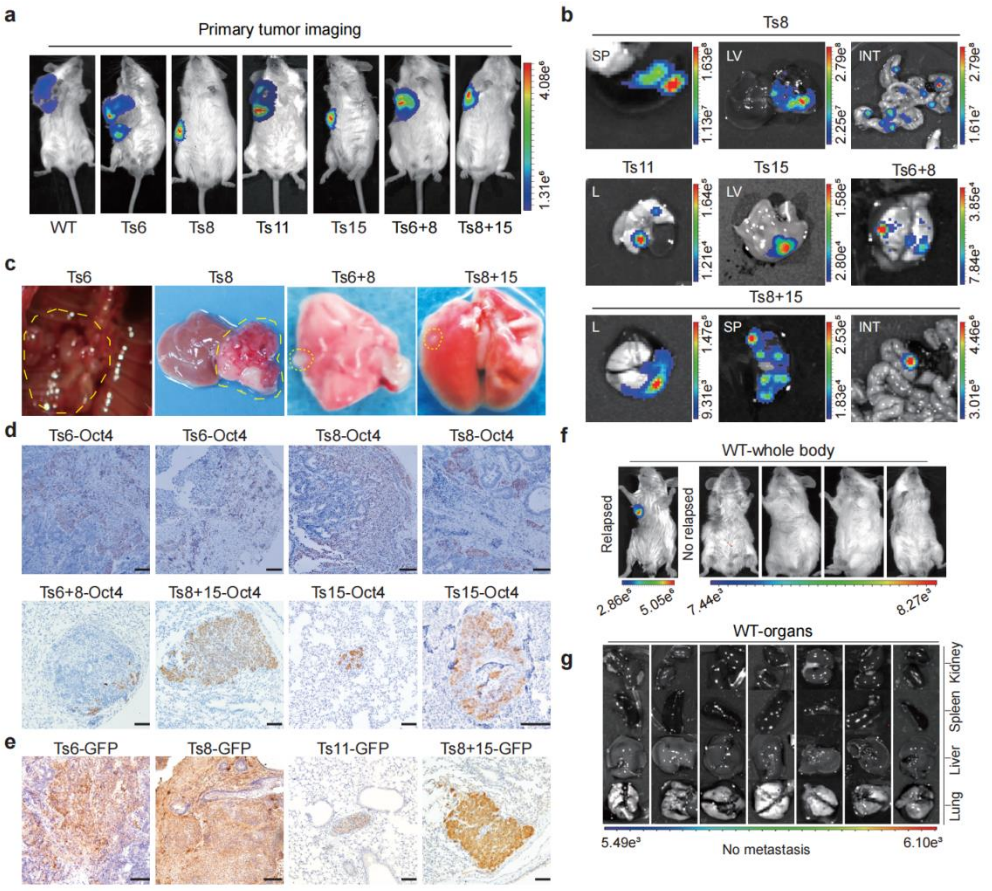

Supplementary Fig. 3 | Multi-organ metastases were observed in mice bearing aneuploid teratomas.

a *In vivo* imaging of primary teratomas in WT and aneuploid tumor-burden mice. The rainbow gradient bar indicates the photon flux. WT, wild-type. Ts, trisomy. **b** *Ex vivo* bioluminescent imaging of organs with metastases from aneuploid ESCs. SP, spleen. LV, liver. INT, intestine. L, lung; **c** Images of metastatic lesions (the yellow dotted circles indicate metastases. Ts6, Mediastinal; Ts8, liver; Ts6+8, lung lobe; Ts8+15, lung). **d** IHC staining to detect Oct4 expression in lung metastases from Ts6, Ts8, Ts6+8, Ts8+15 and Ts15 teratoma-bearing mouse. Scale bars, 100 µm. **e** IHC experiment to detect GFP expression in Ts6, Ts8, Ts11 and Ts8+15 teratoma-bearing mouse lung metastases. Scale bars, 100 µm. **f** Whole-body bioluminescent imaging of mice bearing relapsed WT tumors. **g** *Ex vivo* bioluminescent imaging of multiple organs from mice bearing WT teratomas.

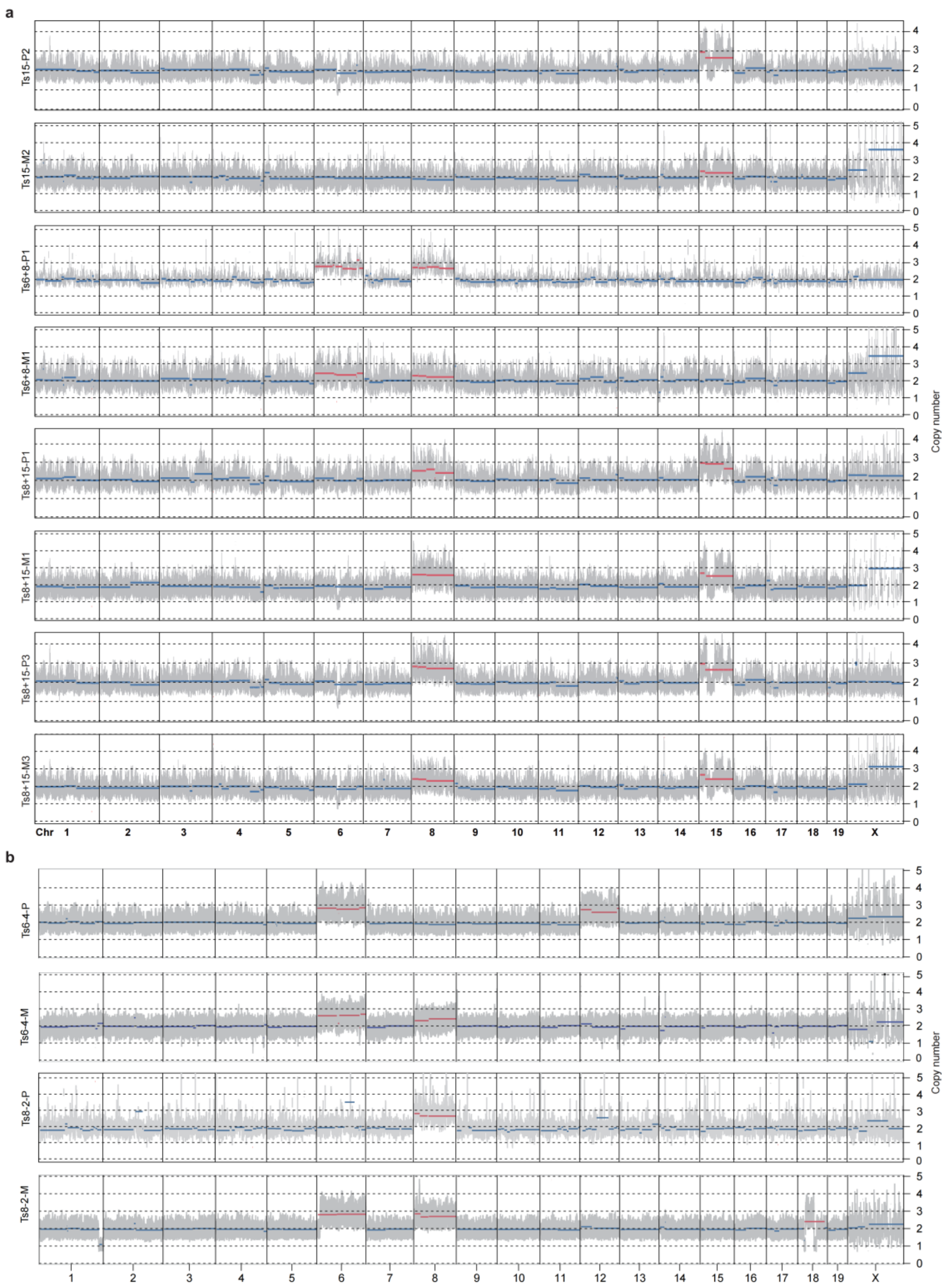

Supplementary Fig. 4 | CNV patterns between paired primary teratomas and metastases.

Chromosome plot indicating CNVs in paired primary teratoma (P) and metastatic (M) samples. **a** Similar CNV patterns between P and M samples. **b** Single-chromosome gains in Ts6 or Ts8 metastases. WT ESCs was used as a control. CNVs were analyzed using Sequenza. A gray bar indicates the interquartile range. The thick horizontal lines indicate segmented values. Trisomic chromosomes are indicated in red. Chr, chromosome. Ts, trisomy.

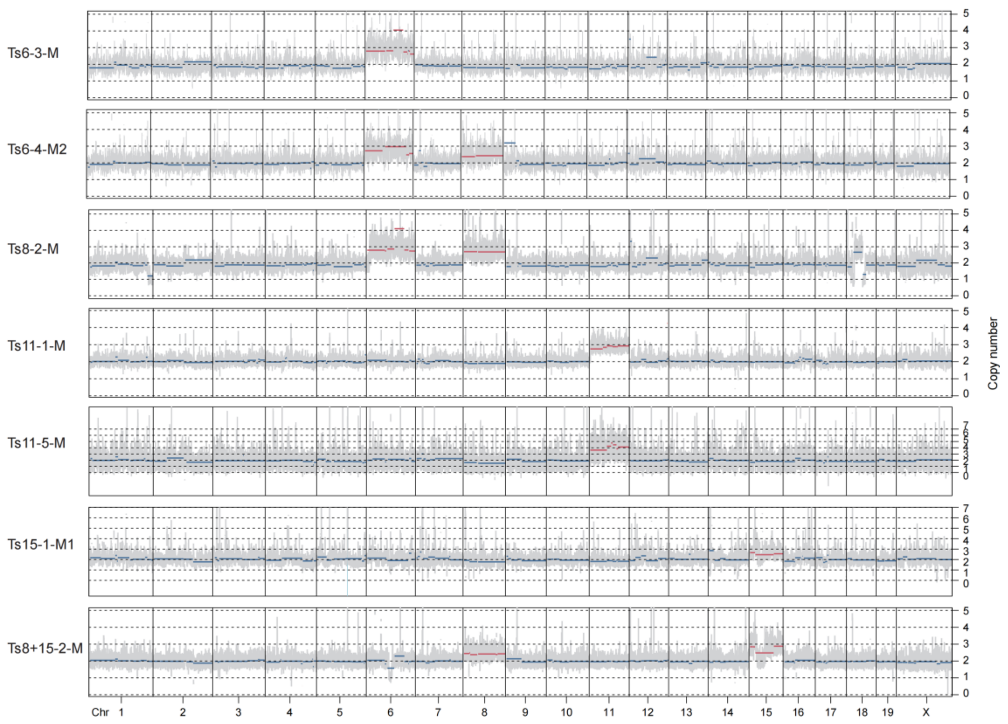

Supplementary Fig. 5 | CNVs detected in WES samples.
Chromosome plot indicating the CNVs of the metastatic (M) samples with each type of aneuploidy. WT ESCs was used as a control. CNVs were analyzed using Sequenza. A gray bar indicates the interquartile range. The thick horizontal lines indicate segmented values. Trisomic chromosomes are indicated in red. Chr, chromosome. Ts, trisomy.

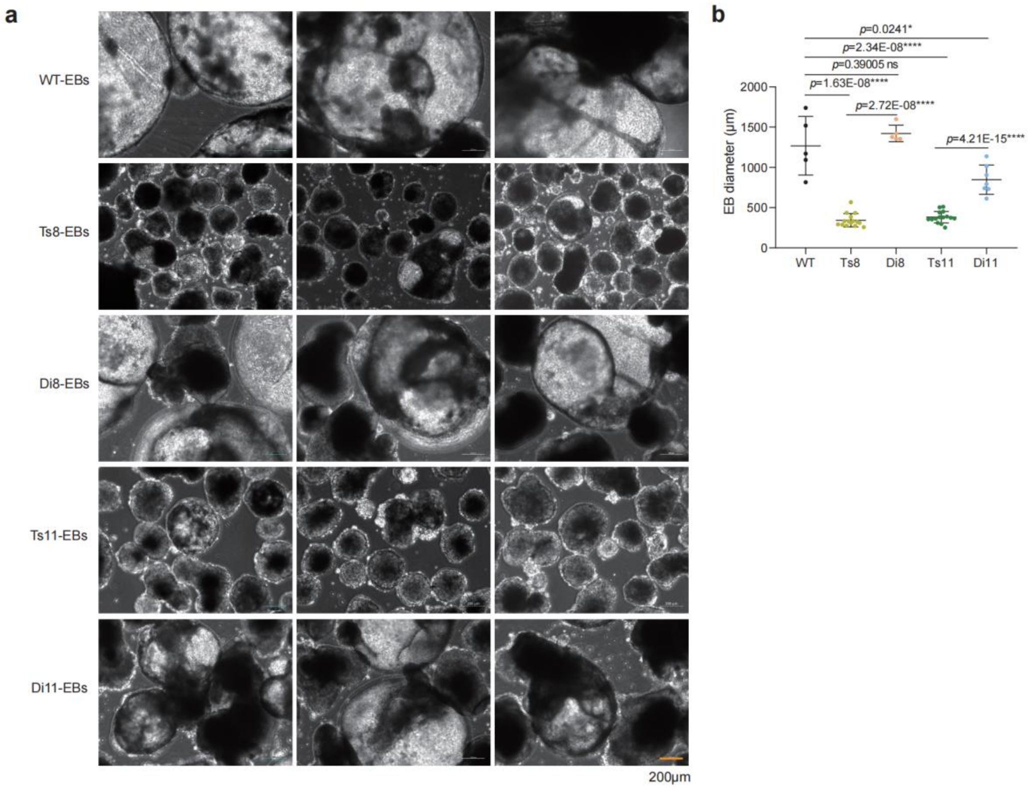

Supplementary Fig. 6 | Trisomy correction rescued the EB formation defects of aneuploid ESCs.

a morphologies of EB formation. Images show EBs from WT, Ts11, Ts8 and the isogenic diploid mESCs (named Di8 and Di11, respectively). Scale bars, 200 μm. **b** Quantification of EB diameters (WT, Di8, n=5; Di11, n=7; Ts8, Ts11, n=15). Ts, trisomy. Di, diploid. Error bars, ± SD. ns, not significant. *P* values were calculated using a two-tailed *t* test, see also Source Data File.

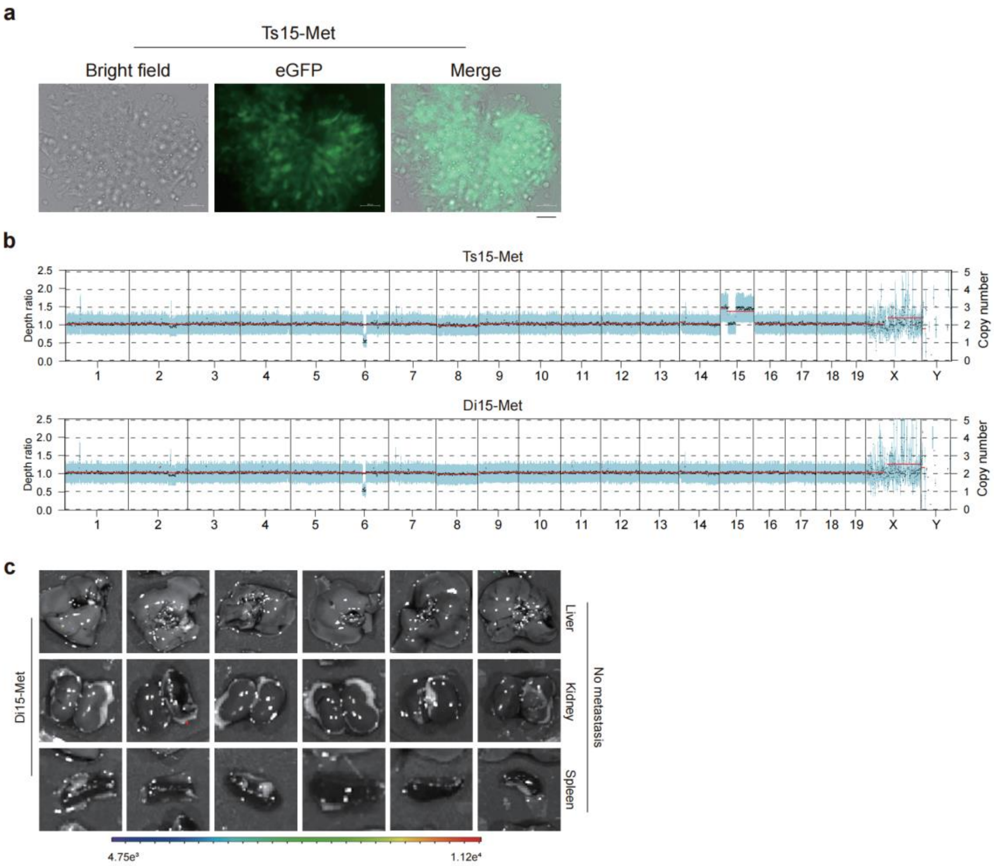

Supplementary Fig. 7 | Trisomy correction of metastatic cell lines.

a Bright-field and fluorescent images of metastatic cells (named Ts15-Met) isolated from Ts15 metastatic mouse. Scale bars, 100 μm. **b** Chromosome plot indicating the CNVs of the metastatic cells (Ts15-Met) and isogenic diploid cells (Di15-Met). WT ESCs was used as a control. CNVs were analyzed using Sequenza. Ts, trisomy; Di, diploidy. **c** BLI imaging of SCID mice injected with Di15-Met (n=6) cells lasted for about one month. The rainbow gradient bar shows the range of photon flux. Di, diploidy.

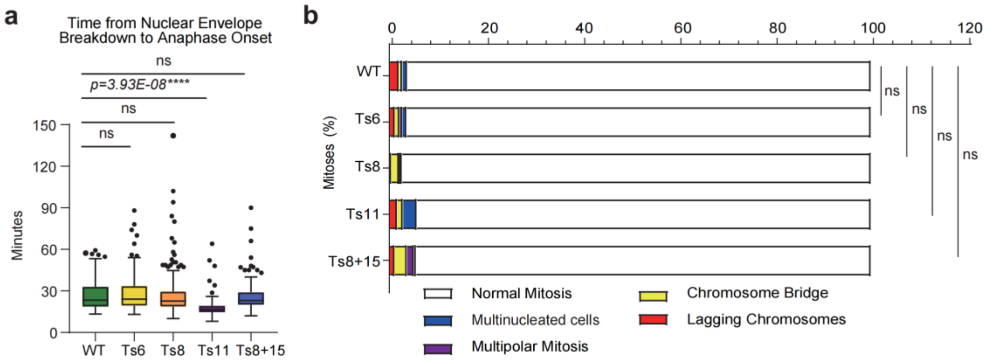

Supplementary Fig. 8 | Comparable CIN levels between WT and aneuploid ESCs. **a** Statistical analysis of the mitotic time (from envelope breakdown to anaphase onset) in WT and aneuploidies. Error bars, ±SD; the data come from three independent experiments (n > 60). *P* values were calculated using a two-tailed *t test*, see also Source Data File. **b** Quantification of mitotic error rates in WT and aneuploid ESC lines. the data come from three independent experiments (n > 60). *P* values were calculated using a two-tailed *t test*, see also Source Data File.

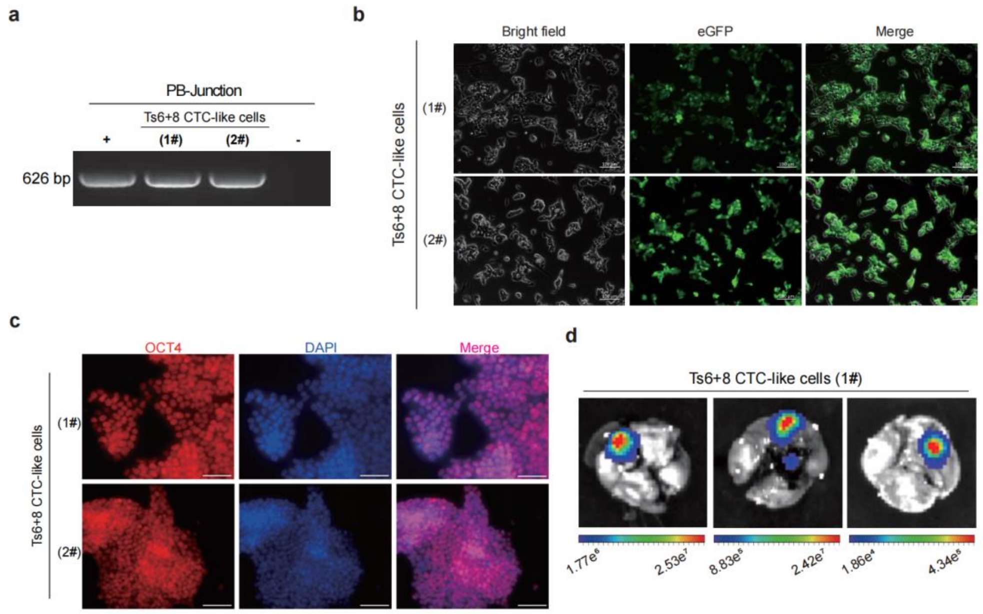

Supplementary Fig. 9 | Characteristics of CTC-like cells isolated from peripheral blood cells.

a PCR amplification of the PB junction to determine the origin of CTC-like cells isolated from Ts6+8 teratoma-bearing mice. PB, *piggyBac*. +, positive control. -, negative control. CTC, circulating tumor cells. See also Source Data File. **b** Bright-field and eGFP images of CTC-like cells. Scale bars, 100 µm. **c** Ts6+8 (1# and 2#) CTC-like cells expressed high levels of Oct4, as shown by immunofluorescence staining. Scale bars, 50 µm. **d** BLI images of metastatic organs (lung) in mice injected with Ts6+8 (1#) CTC-like cells via the tail vein (n=5). The rainbow gradient bar shows the photon flux.

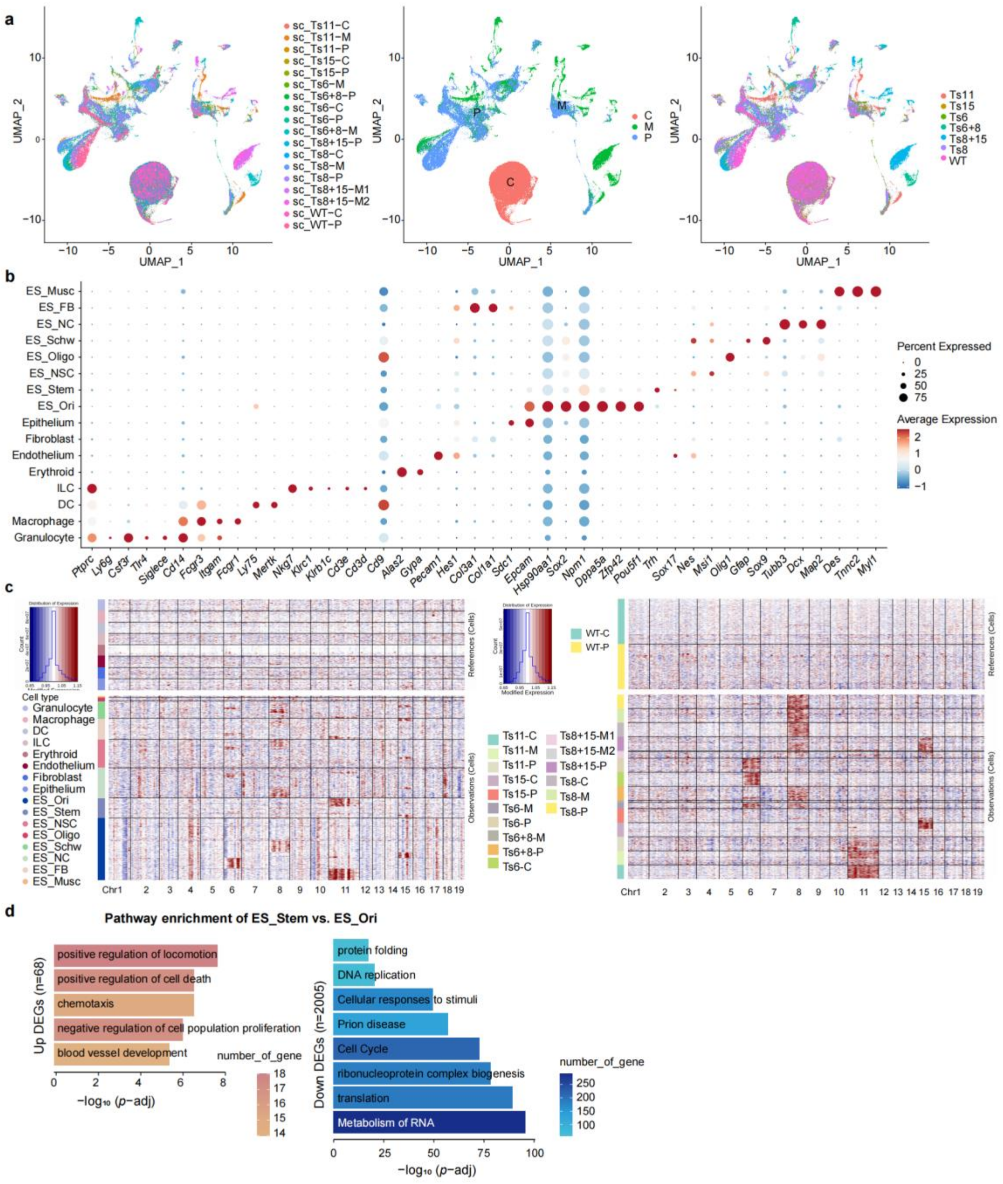

Supplementary Fig. 10 | ScRNA-seq analysis of integrated WT and aneuploid samples.

a UMAP plot displaying the batch effect of integrated scRNA-seq data for WT and aneuploid ESCs (designated WT-C, Ts6-C, Ts8-C, Ts11-C, and Ts15-C, respectively), WT and aneuploid primary teratomas (designated Ts6-P, Ts8-P, Ts11-P, Ts15-P, Ts6+8-P, and Ts8+15-P, respectively) and aneuploid metastases (Ts6-M, Ts8-M, Ts11-M, Ts6+8-M, Ts8+15-M1, and Ts8+15-M2, respectively). Left, sample ID; middle, sample origins including ESCs (C), primary teratomas (P) and metastases (M); right, samples derived from aneuploid or WT samples. **b** Dot plot showing characteristic cell markers of different cell clusters based on integrated scRNA-seq data. **c** InferCNV-detected CNVs of all annotated 16 clusters (Left) and 8 clusters derived from WT and aneuploid ESCs (Right). **d** Pathways enriched in ES_Stem compared with ES_Ori.

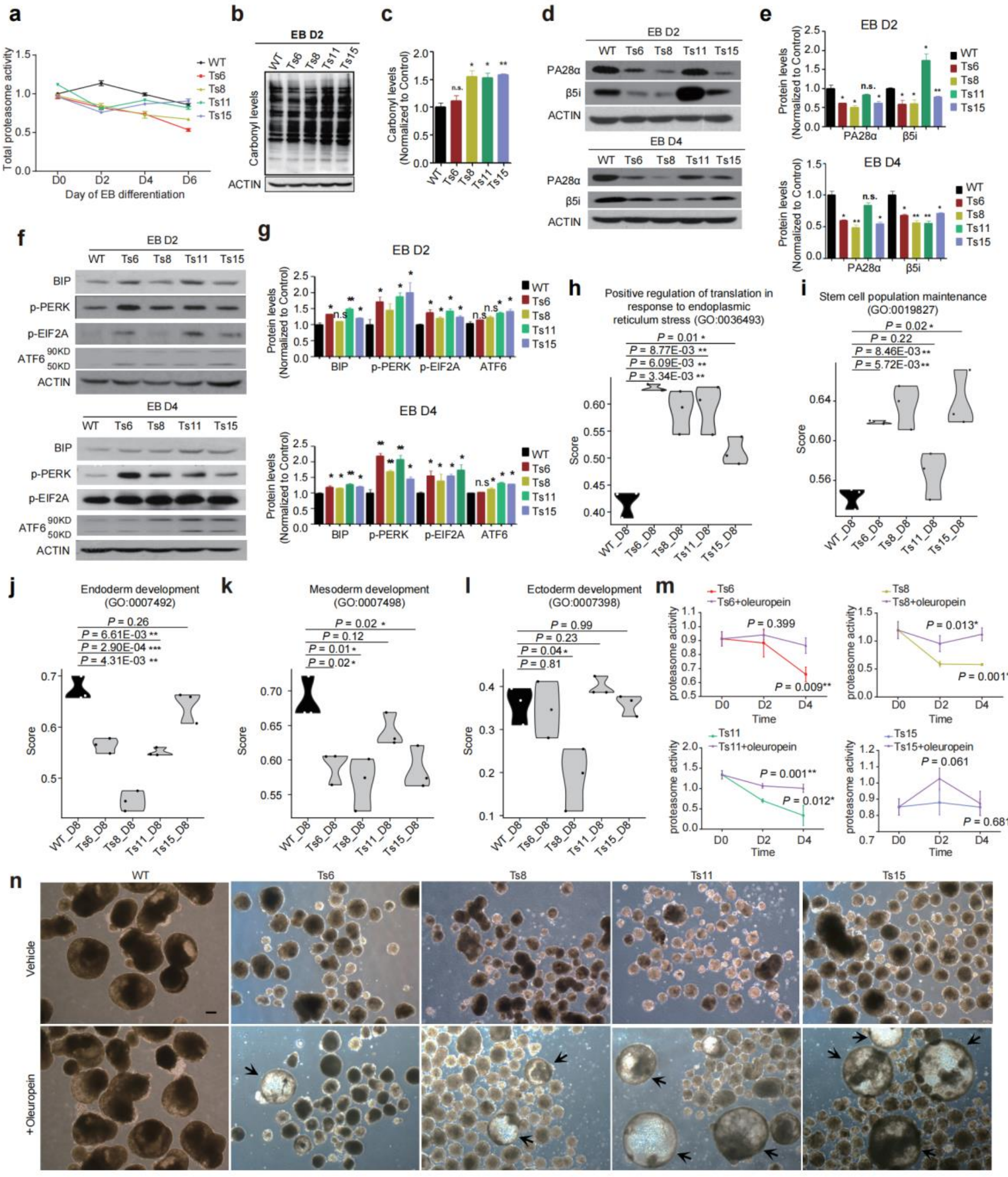

Supplementary Fig. 11 | Aneuploid EBs displayed insufficient proteasome activity and overactivated ER stress.

a Evaluation of proteasome activity of ES cells during EB differentiation. Mean value of undifferentiated WT cells was set to 1.0. Error bars, ±SD, results are quantified with analysis from three independent experimental replicates (n=3). See also Source Data File. **b** Protein carbonyl levels on differentiation day 2 (D2) of WT and aneuploid ES cells. **c** Carbonyl levels were quantified by normalizing band intensities of aneuploid ESCs to WT (n=3). *P < 0.05, **P < 0.01. n.s, not significant. **d** Western blot showing the expression levels of proteasome subunits, including PA28α and β5i, on EB differentiation D2 and D4. See also Source Data File. **e** Quantification of the expression levels of proteasome subunits (PA28α and β5i) on EB differentiation D2 and D4. *P < 0.05, **P < 0.01. n.s, not significant. WT, wild-type; Ts, trisomy. **f** Western blot analysis of the expression of ER stress-associated genes BIP, p-PERK, p-EIF2A, and ATF6 (90 KD: precursor ATF6α and its processed active form-50 KD) on EB

differentiation D2 and D4. See also Source Data File. **g** Quantification of the expression levels of ER stress-associated genes on EB differentiation D2 and D4. **P* < 0.05, ** *P* < 0.01. n.s, not significant. **h** Positive regulation of translation in response to endoplasmic reticulum stress (GO: 0036493) between WT and aneuploid EBs (on day 8). **i** Stemness score evaluation between WT and aneuploid EBs (on day 8). Violin plot showing the scores of endoderm development (**j**), mesoderm development (**k**) and ectoderm development (**l**) gene sets in differentiated EBs (on day 8). The stemness gene set consists of *Pou5f1*, *Nanog*, *Zfp42*, *Dppa5a*. *P* values were calculated using a two-tailed *t* test. Data come from three independent biological replicates. **m** Proteasome activity during EBs formation on D0~D4. Error bars, ±SD. *P* values were calculated using a multiple *t* test, see also Source Data File. Data come from three independent biological replicates. **n** *In vitro* EB differentiation of WT and aneuploid ESCs with or without Oleuropein treatment. Black arrows point to the representative large cystic structures of differentiated EBs. Scale bars, 200 μm.

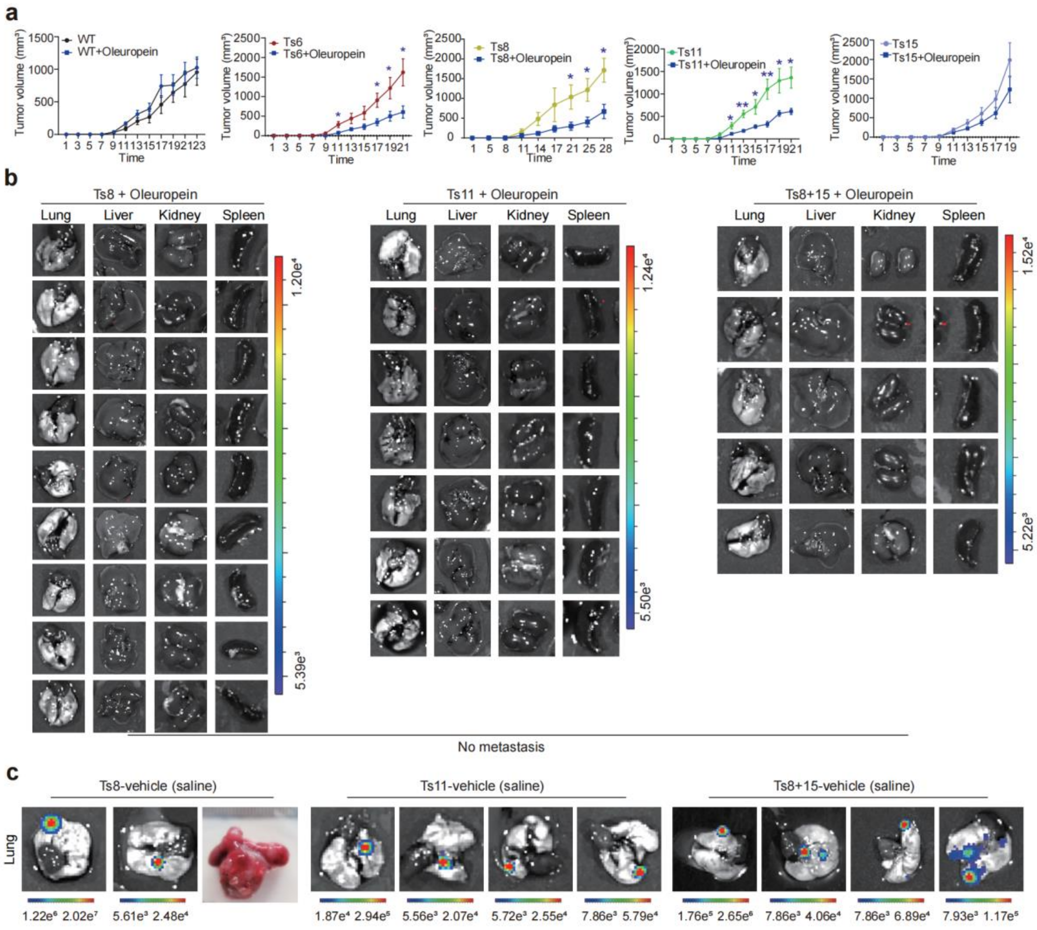

Supplementary Fig. 12 | Proteasome activator Oleuropein can effectively inhibit aneuploid teratoma metastasis.

a Teratoma growth curves of aneuploid teratomas treated with vehicle or Oleuropein (n=8). Error bars, ± SEM. *P* values of each timepoint were calculated using a two-tailed *t* test, see also Source Data File. Eight teratoma sites per group. **b** BLI imaging of organs derived from trisomic teratoma-bearing mice treated with Oleuropein. The rainbow gradient bar represents the photon flux. Number of mice treated with oleuropein (Ts8, n=10; Ts11, n=8; Ts8+15, n=6). **c** BLI imaging of organs derived from trisomic teratoma-bearing mice treated with vehicle (saline). The rainbow gradient bar represents the photon flux. It is noticed that there was one mouse (dead before BLI imaging) of Ts8-vehicle group observed obvious metastases in lung and the bright-field picture was shown. Number of mice treated with saline (Ts8, n=6; Ts11, n=8; Ts8+15, n=7)

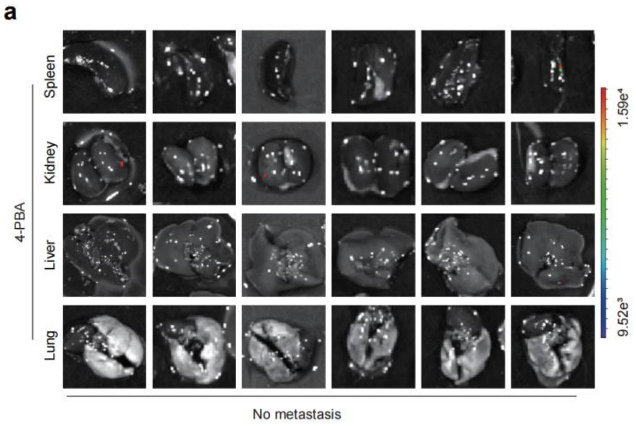

b

Treatment	Met / Total	Sites
4-PBA	0 / 6	-
Vehicle	4 / 7	Lung

Supplementary Fig. 13 | ER stress inhibitor 4-PBA can effectively repress aneuploid teratoma metastasis.

a BLI imaging detected none of the metastatic signals in organs derived from trisomic teratoma-bearing mice treated with 4-PBA. **b** summary of metastatic efficiency of teratoma-bearing mice treated with 4-PBA (Ts8+15, n=6) or vehicle (Ts8+15, n=7).

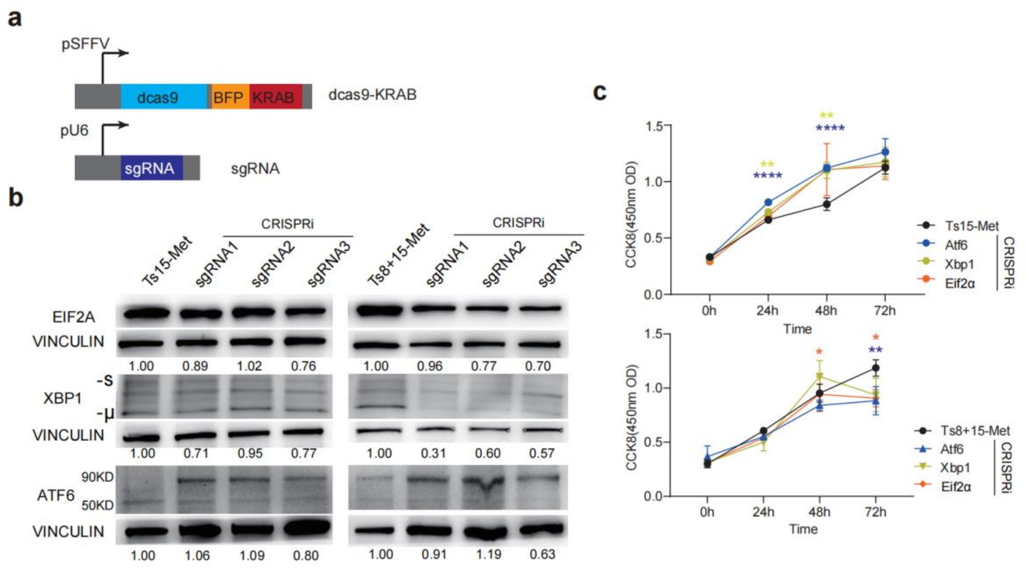

Supplementary Fig. 14 | UPR genes knockdown can effectively repress aneuploid teratoma metastasis.

a CRISPRi plasmids used this study. **b** UPR genes knockdown efficiency demonstrated by western blot experiment and quantified by using image J software. Of them, XBP1 (processed active form: XBP1-s); ATF6 (processed active form: ATF6-50KD). See also Source Data File. **c** Cell proliferation of different cell lines detected by CCK8. *P*-adj values were calculated and adjusted using two-tailed *t* test, see also Source Data File. ns, not significant.

Supplementary table 1. Details of mice bearing teratomas derived from single trisomies.

Aneuploidy	Number	IVIS (sites)	HE (sites)	PCR	Timeline	Sequencing
Ts6	1#	Lung	-	-	61 Days	-
	2#	-	Lung	√	56 Days	-
	3#	-	Liver*	√	44 Days	WGS/WES
	4#	-	Lung*	√	44 Days	WGS/WES/scRNA-seq (Met)
	5#	Lung	-	-	97 Days	-
Ts8	1#	Lung	Lung	√	93 Days	WGS/scRNA-seq (Met)
	2#	Liver*	Liver*	√	58 Days	WGS/WES
	3#	Lung	-	-	65 Days	WGS
	4#	Lung	-	-	88 Days	-
	5#	Lung	-	√	91 Days	-
Ts11	1#	Lung	-	√	121Days	WGS/WES
	2#	-	Lung	-	89 Days	-
	3#	-	Lung	√	78 Days	-
	4#	Lung	-	-	75 Days	-
	5#	-	-	-	92 Days	WES/scRNA-seq (Met)
	6#	-	Lung	-	90 Days	-
	8#	-	Lung	-	76 Days	-
	9#	Lung	-	-	44 Days	-
	10#	-	-	-	42 Days	-
	11#	Lung	-	-	61 Days	-
	12#	Lung	-	-	101 Days	-
	13#	Heart	-	-	101 Days	-
Ts15	1#	Lung	Lung	√	79 Days	WGS/WES
	2#	Lung	-	√	115 Days	WGS
	3#	-	Lung	-	58 Days	-
	4#	-	Lung	-	63 Days	-

*: multi-organ metastases were observed
Ts6-3#: liver, kidney, intestine

Ts6-4#: lung, mediastinal
Ts8-2#: liver, kidney, intestine
KID: kidney; INT: intestine; L: lung; STO: stomach.

1

Supplementary table 2. Details of mice with double trisomies analyzed in this study.

Aneuploidy	Number	IVIS	HE	PCR	Timeline	Sequencing
Ts6+8	1#	Lung	-	√	81 Days	WGS/WES
	2#	Lung	-	-	103 Days	WGS
	3#	Lung	-	√	80 Days	-
	4#	-	-	√	87 Days	-
	5#	-	Lung	-	56 Days	-
	6#	Lung	-	-	99 Days	-
	7#	-	-	√	65 Days	-
	8#	Lung		√	68 Days	-
	9#	-	-	√(CTC-1#)	67 Days	WGS
	10#	-	-	√(CTC-2#)	70 Days	WGS
	11#	Lung	-	√	97 Days	-
	12#	Lung	-	√	93 Days	scRNA-seq (Met)
Ts8+15	1#	KID/INT	-	√	99 Days	WGS
	2#	Lung	Lung	√	87 Days	WGS/WES
	3#	Lung	-	√	99 Days	WGS
	4#	L/STO	-	-	97 Days	-
	5#	Lung	-	√	99 Days	-
	6#	Lung	-	-	84 Days	-
	7#	Lung	-	-	106 Days	-
	8#	Lung	-	-	75 Days	-
	9#	Lung	-	-	75 Days	-
	10#	Lung	-	√	65 Days	scRNA-seq (Met)
	11#	Spleen	-	√	104 Days	-
	12#	Lung	-	√	81 Days	scRNA-seq (Met)
	13#	-	-	√	86 Days	-

14#	Lung	-	-	61 Days	-
15#	Lung	-	-	65 Days	-
16#	Lung	-	-	65 Days	-
17#	Lung	-	-	69 Days	-

Supplementary table 3. Markers used for identifying cell clusters based on scRNA-seq data

Cell clusters	Marker genes
Granulocyte	*Ptprc*, *Ly6g*, *Csf3r*
Macrophage	*Ptprc*, *Itgam*, *Fcgr1*
DC	*Ptprc*, *Mertk*, *Ly75*
ILC	*Ptprc*, *Nkg7*, *Cd3d*
Erythroid	*Alas2*, *Gypa*
Endothelium	*Pecam1*, *Hes1*
Fibroblast	*Col3a1*, *Col1a1*
Epithelium	*Sdc1*, *Epcam*
ES_Ori	*Dppa5a* hi, *Pou5f1* hi, *Epcam*
ES_Stem	*Dppa5a* mid, *Pou5f1* mid, *Trh*
ES_Mus	*Des*, *Tnnc2*, *Myl1*
ES_NSC	*Nes*, *Msi1*, *Sox9* mid
ES_Oligo	*Olig1*
ES_Schw	*Sox9*, *Gfap*
ES_NC	*Tubb3*, *Map2*
ES_FB	*EGFP*, *Col3a1*, *Col1a1*

Hi, higher level of expression; mid, middle level of expression.

Supplementary table 4. List of primers used in this study.

Name	Sequence (5′-3′)
PB3'-1	TAAATAAACCTCGATATACAGACCGATAAA
PB3'-2	ATATACAGACCGATAAAACACATGCGTCAA
PB3'-seq	TTTTACGCATGATTATCTTTAACGTACGTC
PB5'-1	CAAAATCAGTGACACTTACCGCATTGACAA
PB5'-2	CTTACCGCATTGACAAGCACGCCTCACGGG
PB5'-seq	TTAGAAAGAGAGAGCAATATTTCAAGAATG
Ts11-F	ATGGAAACGAGGAAGATGGTTGGAC
Ts11-R	TGCTTTATAGCCATTCAGACCCGAG
Ts15/Ts8+15-F	CCTGACTTCTATGGAGTGTTGCCT
Ts15/Ts8+15-R	GAATGGGGTAACTTTCATGTGCTG
Ts8/Ts6+8-F	CAAGTACCTAAAAAGTCCCACGCA
Ts8/Ts6+8-R	GAGGCTTTATATTCTGGCTCGGTA
Ts6-F	AATCACATTGTTCCCAGTTACCCA
Ts6-R	CGAGTAGCCATGATCGCTGTTATT
β-gal-F	GCGTTGGAGTGACGGCAGTTAT
β-gal-R	ACAGCGGATGGTTCGGATAATG
Ts6-1st-F (Nested PCR)	TAGACCATGAGATAGGATACAGGTA
Ts6-1st-R (Nested PCR)	CTTCAAGTCACAGAAGTACAAGTAT
Ts6-2nd-F (Nested PCR)	GGATACAGGTAAATAAGAAGGCTAG
Ts6-2nd-R (Nested PCR)	GAAGTACAAGTATGATACAGACCAC
EGFP-LUC-F	ACAAGCAGAAGAACGGCATCAA
EGFP-LUC-R	GCGAAATGCCCATACTGTTGAG

Supplementary table 5. Reagents and kits used in this study.

Name	Cat. No	Source
Fialuridine	SML0632	Sigma
Demecolcine Solution	D1925	Sigma
SlowFade Gold with DAPI	S36939	Invitrogen
Hexadimethrine bromide	H9268	Sigma
Matrigel	356234	Corning/BD
D-Luciferin Potassium Salt Bioluminescent Substrate	122799	PerkinElmer
Mouse 1× Lymphocyte Separation Medium	7211011	DAKEWE
2,2,2-Tribromoethanol	T48402	Sigma
Plasmid Maxi Kit	12162	QIAGEN
FFPE Tissue Kit	56404	QIAGEN
Genomic DNA Mini Kit	K1820-01	Invitrogen
Tumor Dissociation Kit, mouse	130-096-730	MACS

2
