# Supplementary material for: Aneuploid embryonic stem cells drive teratoma metastasis
Source: Nat Commun. 2024 Feb 5;15:1087. doi: 10.1038/s41467-024-45265-4 (PMC10844504; doi:10.1038/s41467-024-45265-4)
Supplement: Supplementary file 3 — Description of Additional Supplementary Files [file 41467_2024_45265_MOESM3_ESM.pdf]

Description of Additional Supplementary Files

Supplementary Movie 1: Time-lapse fluorescence microscopy recorded the normal cell division of a Ts11 mES cell.

Supplementary Movie 2: Time-lapse fluorescence microscopy recorded a Ts6 mES cell division error-the chromosome bridge.

Supplementary Movie 3: Time-lapse fluorescence microscopy recorded chromosome segregation of Ts8+15 mES cells. One of them underwent multipolar mitosis.
