# Supplementary material for: Aneuploid embryonic stem cells drive teratoma metastasis
Source: Nat Commun. 2024 Feb 5;15:1087. doi: 10.1038/s41467-024-45265-4 (PMC10844504; doi:10.1038/s41467-024-45265-4)
Supplement: Supplementary file 7 — Reporting Summary [file 41467_2024_45265_MOESM7_ESM.pdf]

nature portfolio

Corresponding author(s): Yue Huang, Fan Bai

Last updated by author(s): Dec 10, 2023

Reporting Summary

Nature Portfolio wishes to improve the reproducibility of the work that we publish. This form provides structure for consistency and transparency in reporting. For further information on Nature Portfolio policies, see our Editorial Policies and the Editorial Policy Checklist.

Statistics

For all statistical analyses, confirm that the following items are present in the figure legend, table legend, main text, or Methods section.

n/a	Confirmed	
☐	☒	The exact sample size (*n*) for each experimental group/condition, given as a discrete number and unit of measurement
☐	☒	A statement on whether measurements were taken from distinct samples or whether the same sample was measured repeatedly
☐	☒	The statistical test(s) used AND whether they are one- or two-sided<br>*Only common tests should be described solely by name; describe more complex techniques in the Methods section.*
☒	☐	A description of all covariates tested
☒	☐	A description of any assumptions or corrections, such as tests of normality and adjustment for multiple comparisons
☐	☒	A full description of the statistical parameters including central tendency (e.g. means) or other basic estimates (e.g. regression coefficient) AND variation (e.g. standard deviation) or associated estimates of uncertainty (e.g. confidence intervals)
☐	☒	For null hypothesis testing, the test statistic (e.g. *F*, *t*, *r*) with confidence intervals, effect sizes, degrees of freedom and *P* value noted<br>*Give P values as exact values whenever suitable.*
☒	☐	For Bayesian analysis, information on the choice of priors and Markov chain Monte Carlo settings
☒	☐	For hierarchical and complex designs, identification of the appropriate level for tests and full reporting of outcomes
☒	☐	Estimates of effect sizes (e.g. Cohen's *d*, Pearson's *r*), indicating how they were calculated

Our web collection on statistics for biologists contains articles on many of the points above.

Software and code

Policy information about availability of computer code

Data collection	Imaging data collection: SmartCapture 3, DMi8, Nanozoomer, Hamamatsu, ZEN3.1 Blue edition, ZEISS Celldiscoverer 7<br>Bioluminescence data collection: PerkinElmer IVIS system<br>Raw data collection of sequencing data: PRJNA790979

Data analysis	Graphpad Prism (v8.1.0)<br>R (v 3.6)<br>fastp (v 0.20.0)<br>bwa (v 0.7.17-r1188)<br>SAMtools (v 1.7)<br>GATK toolkit (v 3.8-0-ge9d806836)<br>vep (v 101.0)<br>vcf2maf (v 1.6.21)<br>maftools (v 2.6.05)<br>sequenza-utils (v 3.0.0)<br>Sequenza R package (v 3.2.0)<br>Cell Ranger (v 3.1.0)<br>Seurat (v 4.0.1)<br>Metascape (Online tools: https://metascape.org/gp/index.html)<br>inferCNV (v 1.6.0)<br>Monocle3 (v 1.0.0)

For manuscripts utilizing custom algorithms or software that are central to the research but not yet described in published literature, software must be made available to editors and reviewers. We strongly encourage code deposition in a community repository (e.g. GitHub). See the Nature Portfolio guidelines for submitting code & software for further information.

Data

Policy information about availability of data

All manuscripts must include a data availability statement. This statement should provide the following information, where applicable:

- Accession codes, unique identifiers, or web links for publicly available datasets
- A description of any restrictions on data availability
- For clinical datasets or third party data, please ensure that the statement adheres to our policy

All codes are available at https://github.com/xds-github/Aneuploidy-drives-teratoma-metastasis. All sequencing data are available at SRA database of NCBI.

Human research participants

Policy information about studies involving human research participants and Sex and Gender in Research.

Reporting on sex and gender	
Population characteristics	
Recruitment	
Ethics oversight	

Note that full information on the approval of the study protocol must also be provided in the manuscript.

Field-specific reporting

Please select the one below that is the best fit for your research. If you are not sure, read the appropriate sections before making your selection.

☒ Life sciences ☐ Behavioural & social sciences ☐ Ecological, evolutionary & environmental sciences

For a reference copy of the document with all sections, see nature.com/documents/nr-reporting-summary-flat.pdf

Life sciences study design

All studies must disclose on these points even when the disclosure is negative.

Sample size	No statistical methods were used to predetermine sample sizes. Sample size was chosen according to the sdandards of the field (at least 3 independent biological replicates for each condition).
Data exclusions	No data were excluded.
Replication	Experiments were performed using at least 3 independent biological replicates to ensure reproducibility.
Randomization	Experimental groups were not randomized. All experiments were performed with appropriate positive and negative controls. For teratoma experiment, mice were randomized into each group.

Blinding | Investigators were not blinded to group allocation, but were blinded to data collection and analysis. All experiments were performed and analyzed using unbiased methodology.

Behavioural & social sciences study design

All studies must disclose on these points even when the disclosure is negative.

Study description

Research sample

Sampling strategy

Data collection

Timing

Data exclusions

Non-participation

Randomization

Ecological, evolutionary & environmental sciences study design

All studies must disclose on these points even when the disclosure is negative.

Study description

Research sample

Sampling strategy

Data collection

Timing and spatial scale

Data exclusions

Reproducibility

Randomization

Blinding

Did the study involve field work? ☐ Yes ☐ No

Field work, collection and transport

Field conditions

Location

Access & import/export

Disturbance

Reporting for specific materials, systems and methods

We require information from authors about some types of materials, experimental systems and methods used in many studies. Here, indicate whether each material, system or method listed is relevant to your study. If you are not sure if a list item applies to your research, read the appropriate section before selecting a response.

Materials & experimental systems

n/a	Involved in the study
☐	☒ Antibodies
☐	☒ Eukaryotic cell lines
☒	☐ Palaeontology and archaeology
☐	☒ Animals and other organisms
☒	☐ Clinical data
☒	☐ Dual use research of concern

Methods

n/a	Involved in the study
☒	☐ ChIP-seq
☒	☐ Flow cytometry
☒	☐ MRI-based neuroimaging

Antibodies

Antibodies used

Rabbit polyclonal anti-Atf6 (Abcam, catalogue number ab37149);
Mouse monoclonal anti-Bip (BD, catalogue number 610978);
Rabbit monoclonal anti-p-Perk (Cell Signaling Technology, catalogue number 3179);
Rabbit polyclonal anti-p-eIF2α (Cell Signaling Technology, catalogue number 9721);
Rabbit polyclonal anti-Pa28α (ENZO, catalogue number BML-PW8185);
Rabbit polyclonal anti-β5i (ENZO, catalogue number BML-PW8400);
Goat polyclonal anti-actin (Santa Cruz Biotechnology, catalogue number sc-1616);
Mouse monoclonal anti-Oct3/4 (Santa Cruz Biotechnology, catalogue number sc-5279);
Rabbit monoclonal anti-GFP (Cell Signaling Technology, catalogue number 2956);
Mouse monoclonal anti-Vinculin (Sigma, catalogue number V9131)
Rabbit polyclonal anti-XBP1 (Abcam, catalogue number ab37152)

Validation

All commercially available antibodies were validated by the suppliers through Westernblot.

Eukaryotic cell lines

Policy information about cell lines and Sex and Gender in Research

Cell line source(s)

AB1 ESCs were obtained from Dr. Allan Bradley (University of Cambridge). Aneuploid ESC lines were established in our lab.

Authentication

The mouse ESC identity was confirmed by their self-renewal and pluripotency abilities.

Mycoplasma contamination

All cell lines were routinely tested for mycoplasma contamination by PCR. No contamination was detected.

Commonly misidentified lines
(See ICLAC register)

No commonly misidentified lines were used.

Palaeontology and Archaeology

Specimen provenance

Specimen deposition

Dating methods

☐ Tick this box to confirm that the raw and calibrated dates are available in the paper or in Supplementary Information.

Ethics oversight

Note that full information on the approval of the study protocol must also be provided in the manuscript.

Animals and other research organisms

Policy information about studies involving animals; ARRIVE guidelines recommended for reporting animal research, and Sex and Gender in Research

Laboratory animals

SCID/Beige male mice (6 weeks old) were used in this study.

Wild animals

No wild animals were used in this study.

Reporting on sex | All mice used in this study are male.

Field-collected samples | No field samples were collected in this study.

Ethics oversight | All animal experiments were approved by the Institutional Animal Care and Use Committee of the Chinese Academy of Medical Sciences & Peking Union Medical College. All animal care and experimental methods followed the ARRIVE guidelines for animal experiments.

Note that full information on the approval of the study protocol must also be provided in the manuscript.

Clinical data

Policy information about clinical studies
All manuscripts should comply with the ICMJE guidelines for publication of clinical research and a completed CONSORT checklist must be included with all submissions.

Clinical trial registration

Study protocol

Data collection

Outcomes

Dual use research of concern

Policy information about dual use research of concern

Hazards

Could the accidental, deliberate or reckless misuse of agents or technologies generated in the work, or the application of information presented in the manuscript, pose a threat to:

No	Yes	
☐	☐	Public health
☐	☐	National security
☐	☐	Crops and/or livestock
☐	☐	Ecosystems
☐	☐	Any other significant area

Experiments of concern

Does the work involve any of these experiments of concern:

No	Yes	
☐	☐	Demonstrate how to render a vaccine ineffective
☐	☐	Confer resistance to therapeutically useful antibiotics or antiviral agents
☐	☐	Enhance the virulence of a pathogen or render a nonpathogen virulent
☐	☐	Increase transmissibility of a pathogen
☐	☐	Alter the host range of a pathogen
☐	☐	Enable evasion of diagnostic/detection modalities
☐	☐	Enable the weaponization of a biological agent or toxin
☐	☐	Any other potentially harmful combination of experiments and agents

ChIP-seq

Data deposition

☐ Confirm that both raw and final processed data have been deposited in a public database such as GEO.

☐ Confirm that you have deposited or provided access to graph files (e.g. BED files) for the called peaks.

Data access links
May remain private before publication.

Files in database submission

Genome browser session
(e.g. UCSC)

Methodology

Replicates

Sequencing depth

Antibodies

Peak calling parameters

Data quality

Software

Flow Cytometry

Plots

Confirm that:

☐ The axis labels state the marker and fluorochrome used (e.g. CD4-FITC).

☐ The axis scales are clearly visible. Include numbers along axes only for bottom left plot of group (a 'group' is an analysis of identical markers).

☐ All plots are contour plots with outliers or pseudocolor plots.

☐ A numerical value for number of cells or percentage (with statistics) is provided.

Methodology

Sample preparation

Instrument

Software

Cell population abundance

Gating strategy

☐ Tick this box to confirm that a figure exemplifying the gating strategy is provided in the Supplementary Information.

Magnetic resonance imaging

Experimental design

Design type

Design specifications

Behavioral performance measures

Acquisition

Imaging type(s)

Field strength

Sequence & imaging parameters

Area of acquisition

Diffusion MRI ☐ Used ☐ Not used

Preprocessing

Preprocessing software

Normalization

Normalization template

Noise and artifact removal

Volume censoring

Statistical modeling & inference

Model type and settings

Effect(s) tested

Specify type of analysis: ☐ Whole brain ☐ ROI-based ☐ Both

Statistic type for inference
(See Eklund et al. 2016)

Correction

Models & analysis

n/a	Involved in the study
☐	☐ Functional and/or effective connectivity
☐	☐ Graph analysis
☐	☐ Multivariate modeling or predictive analysis

Functional and/or effective connectivity

Graph analysis

Multivariate modeling and predictive analysis
